# Phytoconstituents with Radical Scavenging and Cytotoxic Activities from *Diospyros shimbaensis*

**DOI:** 10.3390/diseases4010003

**Published:** 2016-01-15

**Authors:** Per Aronsson, Joan J. E. Munissi, Amra Gruhonjic, Paul A. Fitzpatrick, Göran Landberg, Stephen S. Nyandoro, Mate Erdelyi

**Affiliations:** 1 Department of Chemistry and Molecular Biology, University of Gothenburg, Gothenburg SE-412 96, Sweden; aronsson.per.m@gmail.com (P.A.); amragruhonjic@hotmail.com (A.G.); 2 Chemistry Department, College of Natural and Applied Sciences, University of Dar es Salaam, P.O. Box. 35061, Dar es Salaam 0255, Tanzania; 3 Sahlgrenska Cancer Center, University of Gothenburg, Gothenburg SE-405 30, Sweden; paul.fitzpatrick@gu.se (P.A.F.); goran.landberg@gu.se (G.L.); 4 Swedish NMR Center, University of Gothenburg, Gothenburg SE-405 30, Sweden

**Keywords:** *Diospyros shimbaensis*, Ebenaceae, tetralones, naphthoquinoes, radical scavenging, cytotoxicity, MDA-MB-231, DPPH

## Abstract

As part of our search for natural products having antioxidant and anticancer properties, the phytochemical investigation of *Diospyros shimbaensis* (Ebenaceae), a plant belonging to a genus widely used in East African traditional medicine, was carried out. From its stem and root barks the new naphthoquinone 8,8′-oxo-biplumbagin (**1**) was isolated along with the known tetralones *trans*-isoshinanolone (**2**) and *cis*-isoshinanolone (**3**), and the naphthoquinones plumbagin (**4**) and 3,3′-biplumbagin (**5**). Compounds **2**, **4**, and **5** showed cytotoxicity (IC_50_ 520–82.1 μM) against MDA-MB-231 breast cancer cells. Moderate to low cytotoxicity was observed for the hexane, dichloromethane, and methanol extracts of the root bark (IC_50_ 16.1, 29.7 and > 100 μg/mL, respectively), and for the methanol extract of the stem bark (IC_50_ 59.6 μg/mL). The radical scavenging activity of the isolated constituents (**1**–**5**) was evaluated on the 2,2-diphenyl-1-picrylhydrazyl (DPPH) radical scavenging assay. The applicability of the crude extracts and of the isolated constituents for controlling degenerative diseases is discussed.

## 1. Introduction

The genus *Diospyros* (Ebenaceae) comprises of approximately 500 species distributed in the tropical and subtropical regions of both hemispheres. Several *Diospyros* species are known for their timber and edible fruits, e.g., *D*. *kaki*, *D*. *kirkii* [1], and are used in traditional medicine. For example, *D*. *lycioides* is applied against dental infections [2,3], *D*. *maritima* against rheumatism [4,5], *D*. *kirkii* against fever and gonorrhea [6], and *D*. *usambarensis* as antidote against snake bite [6,7,8]. In Tanzania, the root decoctions of *D. fischeri* are taken against stomach aches, chest complaints, gonorrhea, and dry cough. The roots and leaves of *D. usambarensis* are used for the treatment of stomach pain, constipation, rashes, cervical prolapse, epilepsy, malaria, measles, psychiatric disorders, sterility, and joint pain, whereas those of *D. verrucosa* for the treatment of psychiatric disorders and its fruits against asthenia [9]. This extensive traditional medicinal use of the *Diospyros* genus suggests its richness in bioactive secondary metabolites. Accordingly, the isolation of triterpenoids, coumarins, naphthols, phenols, and naphthoquinones from this genus was reported [10,11]. *D*. *shimbaensis*, a small tree native to East Africa, has not yet been phytochemically investigated. It has very limited geographical distribution with a minimal remaining population, and was therefore red-listed by IUCN as an endangered species [12]. In order to support its domestication and to avoid an unfortunate loss of phytochemical information and bioactive compounds that may be of medicinal interest, we have investigated its stem and root barks constituents for radical scavenging and cytotoxic properties.

## 2. Results and Discussion

### 2.1. Isolation and Identification of Compounds

**Figure 1:**
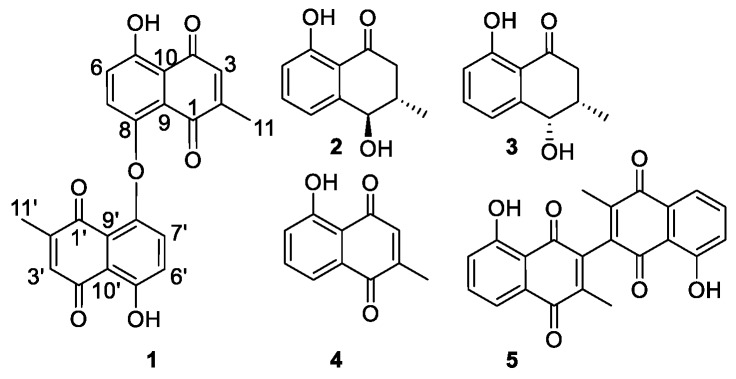
Compounds **1**–**5** isolated from *D*. *shimbaensis*.

The air dried stem bark of *D. shimbaensis* was extracted by soaking in methanol at room temperature for 48 h. Its root bark was extracted consecutively in isohexane, dichloromethane, and methanol, by soaking at room temperature for 48 h each. The methanol extracts of the stem and root barks were separately subjected to purification by high-performance liquid chromatography (HPLC) yielding five compounds (Figure 1), which were identified by NMR spectroscopic and mass spectrometric analyses.

Compound **1** (Figure 1) was obtained as a yellow amorphous solid. Its HR(ESI)MS analysis along with its ^13^C-NMR data indicated the molecular formula C_22_H_15_O_7_ (obs *m*/*z* 391.0892, calcd 391.0818, [M + H]^+^). Two carbonyl groups at δ_C_ 190.5 and δ_C_ 185.2, and eight aromatic carbons (Table 1) were observed in its ^13^C-NMR spectrum, consistent with a 1,4-naphthoquinone skeleton. Its aromatic AB spin system δ_H_ 7.29 (H-6 and H-6′) and δ_H_ 7.20 (H-7 and H-7′) with *J* = 8.6 Hz along with a broad singlet at δ_H_ 12.58 (5-OH and 5′-OH), typical of a hydrogen bonded phenol, were consistent with a 5,8-disubstituted benzene ring of a 1,4-naphthoquinone. The observation of a quartet at δ_H_ 6.80 (H-3 and H-3′) and a doublet at δ_H_ 2.01 (H-11 and H-11′), with *J* = 1.6 Hz, suggested the presence of a quinonoid proton neighboring a methyl group. HMBC correlations of this methyl to C-1/C-1′, C-2/C-2′ and C-3/C-3′were consistent with its placement at C-2/C-2′, which also is in good agreement with the biogenesis of plumbagin-type napthoquinones [13]. The quinonoid H-3/H-3′ proton (δ_H_ 6.81) exhibited HMBC correlations to C-10/C-10′ (δ_C_ 115.5), which carbon also showed correlations to H-6/H-6′ (δ_H_ 7.29), confirming the orientation of the rings of the naphthoquinone skeleton of **1**. HMBC correlations of 5-OH/5′-OH (δ_H_ 12.58) to C-10/C-10′, C-5/C-5′ and C-6/C-6′ confirmed its placement at C-5/C-5′. The carbon C-9/C-9′ (δ_C_ 128.2) was identified based on its HMBC correlation to H-7/H-7′ (δ_H_ 7.20). Most spectroscopic features of **1** were found similar to that of maritinone (8,8′-biplumbagin) [14], lacking a proton at its C-8/C-8′ and its molecular mass indicated that it is a symmetric dimer, connected via an oxygen bridge between its C-8 and C-8′. Based on the above spectroscopic evidence, the isolated compound **1** was identified as the new natural product 8,8′-oxo-biplumbagin.

**Table 1:** ^1^H and ^13^C-NMR data for 8,8′-oxo-biplumbagin (**1**).

Position	δ_H_ (I, *m*, *J* in Hz)	δ_C_	HMBC (^2^*J*, ^3^*J*)
1,1′	-	185.2	-
2,2′	-	150.0	-
3,3′	6.80, (2H, q, 1.6)	134.9	C1, C1′, C10, C10′, C11, C11′
4,4′	-	190.5	-
5,5′	-	161.3	-
6,6′	7.29, (2H, d, 8.6)	124.3	C10, C10′, C5, C5′, C7, C7′, C8, C8′
7,7′	7.20 (2H, d, 8.6)	137.9	C5, C5′, C6, C6′, C8, C8′, C9,C9′
8,8′	-	135.6	-
9, 9′	-	128.2	-
10, 10′	-	115.5	-
11,11′	2.01 (6H, d, 1.6)		C1, C1′, C2, C2′, C3, C3′
5-OH, 5′-OH	12.58 (2H, brs)	-	C10, C10′, C5, C5′, C6, C6′

**Figure 2:**
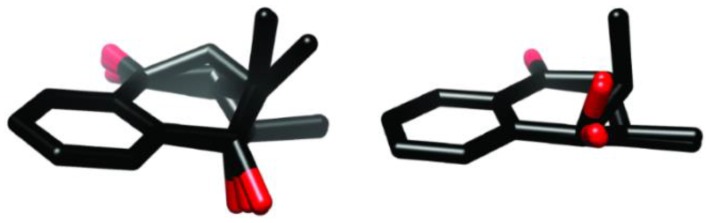
The solution conformations of compounds **2**, to the left, and that of **3**, to the right, as determined by combined theoretical and solution NMR conformational analysis (NAMFIS) for chloroform solution.

Based on their spectroscopic features, compounds **2**–**5** were identified (Supporting Information) as *trans*-isoshinanolone (**2**), *cis*-isoshinanolone (**3**) [15,16,17,18,19,20,21,22,23,24,25], plumbagin (**4**) [26,27,28,29,30], and 3,3′-biplumbagin (**5**) [14,17,25]. Of the five isolated compounds **1**, **4**, and **5** possess rigid planar 1,4-naphthoquinone skeletons whereas the saturated hydroxyl-cyclohexanone ring of **2** and **3** allow some conformational flexibility. In order to determine the solution conformation of the ring of the *cis*- and *trans*-analogues,a NAMFIS analysis was performed [31]. This technique has been successfully applied for the analysis of the conformation and flexibility of natural products [32], peptides [33], and synthetic bioactive substances [34,35]. Theoretically available conformations of **2** and **3** were generated using a Monte Carlo conformational search followed by molecular mechanic energy minimization, resulting in six and eight geometries, respectively. These were used as input conformations for NAMFIS analyses along with the observed NMR parameters ^3^*J*_H1-H2_, ^3^*J*_H2-H3a_, ^3^*J*_H2-H3b_, and NOE distances observed for H2, H3, and H4 using the NOEs observed for vicinal aromatic protons as internal distance reference (2.5 Å). NOESY spectra were acquired for CDCl_3_ solutions at an 800 MHz Bruker Avance III spectrometer (Billerica, MA, USA) equipped with a TCI cryogenic probe, and using 700 ms mixing time and 2.5 s relaxation delay. Further experimental details are given in the Supporting Information. The ensemble analyses of **2** indicated the presence of three major solution conformations distributed in a 1:1:8 ratio, whereas the NAMFIS analysis of **3** revealed two major conformers distributed in a 1:9 ratio, with the energetically most advantaged geometry putting the 2-methyl substituent into equatorial position being favored (Tables S1 and S2 and Figure 2).

### 2.2. Cytotoxicity and Radical Scavenging Activities

The Ebenaceae family is known as a rich source of naphthoquinones, whose quinone core is presumably capable of modulating oxidative biochemical processes [36]. The quinone structural element is common in cancer chemotherapeutic agents, such as doxorubicin, mitomycin C, and mitoxantrone. Accordingly, herbal quinones were reported to possess cytotoxic activity [37,38,39,40,41]. Compounds **2**, **4**, and **5** were therefore assayed for activity against the MDA-MB-231 ER negative breast cancer cell line. Compounds **1** and **3** were only obtained in minute quantities and could therefore unfortunately not be assayed. Whereas **2** showed low toxicity, compounds **4** and **5** were active against the MDA-MB-231 cancer cells (Table 2). It should be noted here that **4** has previously been reported to exhibit anticancer, antileishmanial, antimicrobial, and antituberculotic activities [14,15,28,29,42], which may be exerted via a broad, general cytotoxicity. Accordingly, **4** was reported to cleave mammalian DNA through a topoisomerase II-mediated mechanism *in vitro* [43]. Moreover, in agreement with our above observations DNA-chelation of a hydroxyquinone moiety and iron was previously reported to result in complexes that generate reactive oxygen species (ROS), which may induce single strand breakage and lead to apoptosis [44].

**Table 2:** *In vitro* cytotoxic and radical scavenging activities (RSA) of the crude extracts and isolated constituents from *D. shimbaensis*.

Tested Compound/Extract	Cytotoxicity IC_50_^a^	RSA EC_50_^b^ (µM)
8,8′-Oxo-biplumbagin (**1**)	NT	>25
*trans*-Isoshinanolone (**2**)	>520	>25
*cis*-Isoshinanolone (**3**)	NT	12.5
Plumbagin (**4**)	130.8	>25
3,3′-Biplumbagin (**5**)	82.1	>25
Methanol crude extract from the stem barks	59.6	NT
Isohexane crude extract from the root barks	16.1	NT
Dichloromethane crude extract from the root barks	29.7	NT
Methanol crude extract from the root barks	>100	NT
Dichloromethane crude extract from the leaves	73.0	NT
Methanol crude extract from the leaves	44.7	NT

^a^ IC_50_: half maximal inhibition concentration, is given in μM for pure compounds and μg/mL for crude extracts; ^b^ EC_50_: half maximal effective concentration; NT: not tested.

In a radical scavenging activity (RSA) assay against 2,2-diphenyl-1-picrylhydrazyl (DPPH), the isolated constituents from *D. shimbaensis* showed moderate activity (Table 2 and Table S3) with *cis*-isoshinanolone (**3**) being the most effective. Their moderate RSA may be due to strong intramolecular chelation involving the phenolic hydrogen that is expected to be involved in the antioxidant activity. The observed relatively higher RSA of **3** as compared to its isomer **2** could be associated to their differences in conformation and configuration, which may influence their accessibility to the DPPH radical centre [45]. Generally, DNA damage accelerated by ROS of various types was pointed out as one of the major causes for cancer and degenerative diseases [10]. Also it has been shown that a number of compounds exhibiting antioxidant capabilities can alter cancer cell proliferation, migration, and survival through disruption of the cells redox state. In addition to the potential anti-cancer usage, plant constituents, such as **1**–**5**, with RSA might be deployed for prevention and control of the degenerative diseases by modulating their antioxidant properties [46,47,48,49].

## 3. Experimental Section

### 3.1. General Information

Analytical HPLC was performed using a Hewlett Packard 1050 series instrument (Agilent Technologies Inc, Palo Alto, CA, USA) equipped with a Waters in-line degasser (Waters Corp, Milford, MA, USA) and a Gemini RP 5 μm C18 column (50 × 4.6 mm, Phenomenex, Torrance, CA, USA). Preparative HPLC was run on a Waters 600E HPLC system (Waters Corp) using the Chromulan software with XBridge C-18 column (19 × 150 mm, Waters Corp) using water-methanol eluent mixtures. High-resolution mass spectrometric analyses (Q-TOF-MS) was done by Stenhagen Analyslab AB (Gothenburg, Sweden) using a Micromass Q-TOF micro instrument (Waters Corp) equipped with a lockmass-ESI source with 70 eV ionization voltage. ^1^H and ^13^C-NMR spectra were acquired at room temperature for CDCl_3_ solutions using a Bruker Avance III 800 MHz (^1^H: 799.87 MHz, ^13^C: 201.15 MHz), a Varian Unity 600 MHz (^1^H: 599.77 MHz; ^13^C: 150.83 MHz, Palo Alto, CA, USA), or a Varian VNMR-S 500 MHz (^1^H: 499.58 MHz; ^13^C: 125.71 MHz) spectrometer. Chemical shifts were referenced indirectly to tetramethylsilane via the residual solvent signal (CDCl_3_, ^1^H at 7.26 ppm, ^13^C at 77.16 ppm). Spectra were processed using the software MestReNova (10.1, Mestrelab Research SL, Santiago de Compostela, Spain) and assignment was performed using ^1^H, ^13^C, COSY [50], NOESY [51], HSQC [52], and HMBC [53] spectra. An Agilent 7820A Gas Chromatograph (Agilent Technologies Inc) with an Agilent 5977E MSD MS detector with EI (70 eV) ionization was applied for mass spectrometric analyses. IR spectra were acquired on a PerkinElmer Spectrum One FT-IR (Perkin Elmer Inc., Waltham, MA, USA).

### 3.2. Plant Materials

The stem and root barks of *D*. *shimbaensis* were collected in March 2013 from the Zaraninge Forest Reserve, Bagamoyo District, Pwani region in Tanzania at 300 m altitude. Field identification of the plant species was carried out by Mr. F. M. Mbago of the Herbarium of the Botany Department at the University of Dar es Salaam, where a voucher specimen (FMM 3614) was deposited.

### 3.3. Extraction and Isolation

The air-dried and pulverized stem bark (2 kg) was extracted by soaking twice in methanol for 48 h at room temperature. The filtrate of the extract was concentrated using a rotary evaporator yielding a dark yellowish crude extract (100 g). Portions of this crude (100 mg) were purified by preparative HPLC using a 70:30 to 100:0 water/methanol gradient for 60 min, yielding 8,8′-oxo-bisplumbagin (**1**, 1.6 mg), *trans*-isoshinanolone (**2**, 10.6 mg), *cis*-isoshinanolone (**3**, 1.6 mg), plumbagin (**4**, 40.4 mg), and 3,3′-bisplumbagin (**5**, 1.4 mg).

The air dried and pulverized root bark (1.2 kg) was extracted consecutively in isohexane, dichloromethane, and methanol, by soaking twice for 48 h at room temperature. The filtrates were concentrated on a rotary evaporator yielding 6.8 g (isohexane), 11.2 g (dichloromethane), and 50.6 g (methanol) crude extracts, respectively. The constituents of the methanolic crude extract were isolated by preparative HPLC using a 70:30 to 100:0 water/methanol gradient for 60 min, yielding compounds **2** and **4**.

### 3.4. Cytotoxicity Assay

Cytotoxicity assay was performed on MDA-MB-231 human breast cancer cells using a previously described procedure [54,55]. In short, MDA-MB-231 cancer cells were cultured in Dulbecco’s modified eagle medium (DMEM) supplemented with 10% (*v*/*v*) fetal bovine serum, 2 mM l-glutamine, 100 units/mL penicillin and 100 μg/mL streptomycin at 37 °C in humidified 5% CO_2_. The cells were seeded in 96-well plates at optimal cell density (10,000 cells per well) to ensure exponential growth for the duration of the assay. After a 24 h pre-incubation growth period, the medium was replaced with the experimental medium containing the appropriate drug concentrations or vehicle controls (0.1% or 1.0% *v*/*v* DMSO). After 72 h of incubation, cell viability was measured using the Alamar Blue reagent (Invitrogen Ab, Lidingö, Sweden), according to the manufacturer’s instructions. Absorbance was measured at 570 nm with 600 nm as a reference wavelength. Results were expressed as mean ± standard error for six replicates as a percentage of vehicle control (taken as 100%). Extract or pure compound that showed < 80% viability was considered as a positive hit for cytotoxicity. Such extracts or compounds were further subjected to new cell library testing under serial dilutions to obtain IC_50_.

### 3.5. Radical Scavenging Activities Assay

The method described by Ohnishi *et al.* [56] was used with minor modifications. Test sample solutions of each compound at concentrations of 25, 12.5, 6.25, and 3.13 μM were prepared in methanol (HPLC grade). From each concentration, 1.0 mL sample was added to 2.0 mL of 50 μM DPPH dissolved in methanol. The mixture was allowed to stand at room temperature for 30 min, and the absorbance of the remaining DPPH was then measured at 515 nm. The radical scavenging activity was measured as the decrease of the absorbance of the DPPH radical, expressed as a percentage of the absorbance of the control solution (1.0 mL of methanol mixed with 2.0 mL of 50 μM DPPH solution). Radical scavenging activity was then expressed as effective concentration (EC_50_), thus the concentration of the test compound required for 50% decrease in absorbance as compared to that of the control solution. The percentage of scavenged DPPH was calculated as 100 × (A_DPPH_ − A_Sample_)/A_DPPH_, where A_DPPH_ is the absorbance of the solution containing DPPH but without a test sample and A_Sample_ is the absorbance of the mixture of the test sample and DPPH solution. Experiments were run in triplicate and EC_50_s were determined by plotting the percentage of scavenged DPPH *versus* the initial concentration of each sample.

## 4. Conclusions

Phytochemical analysis of the stem and root barks of *D. shimbaensis* revealed tetralone and naphthoquinone metabolite content, in agreement with these being common chemotaxonomic markers for the genus *Diospyros*. 8,8′-Oxo-biplumbagin is hereby reported for the very first time, whereas the isomeric tetralones *cis-* and *trans*-isoshinalonone and the naphthoquinones plumbagin and 3,3′-biplumbagin are reported for the first time from *D. shimbaensis*. The isolated constituents showed moderate anticancer and radical scavenging activities, which is in line with the traditional medicinal use of the genus and may offer insights applicable in the development of future anticancer agents.

## Supplementary Materials

Supplementary materials are accessible at: http://www.mdpi.com/2079-9721/4/1/3/s1.
