# Supplementary material for: Phytoconstituents with Radical Scavenging and Cytotoxic Activities from Diospyros shimbaensis
Source: Diseases. 2016 Jan 15;4(1):3. doi: 10.3390/diseases4010003 (PMC5456303; doi:10.3390/diseases4010003)
Supplement: Supplementary file 1 [file diseases-04-00003-s001.pdf]

Supplementary Materials: Phytoconstituents with Radical Scavenging and Cytotoxic Activities from *Diospyros shimbaensis*

Per Aronsson [1], Joan J. E. Munissi [2,*], Amra Gruhonjic [1,3], Paul A. Fitzpatrick [3], Göran Landberg [3], Stephen S. Nyandoro [2,*] and Mate Erdelyi [1,4,*]

1. NMR and Mass Spectra of 8,8′-Oxo-biplumbagin (1)

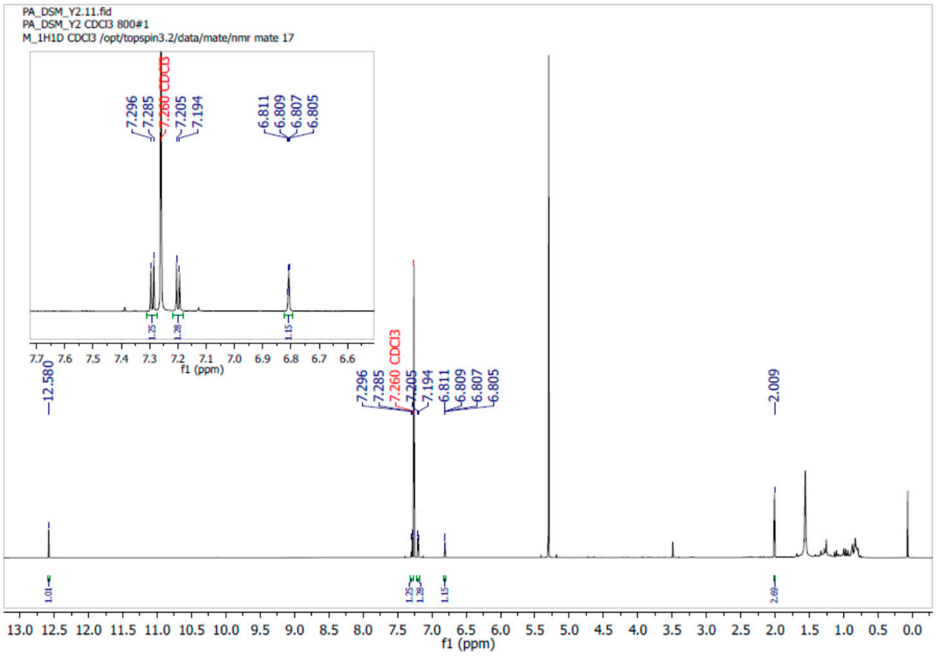

Figure S1. The ^{1}H-NMR spectrum of 8,8′-oxo-biplumbagin (**1**, $CDCl_3$, 25 °C, 799.88 MHz).

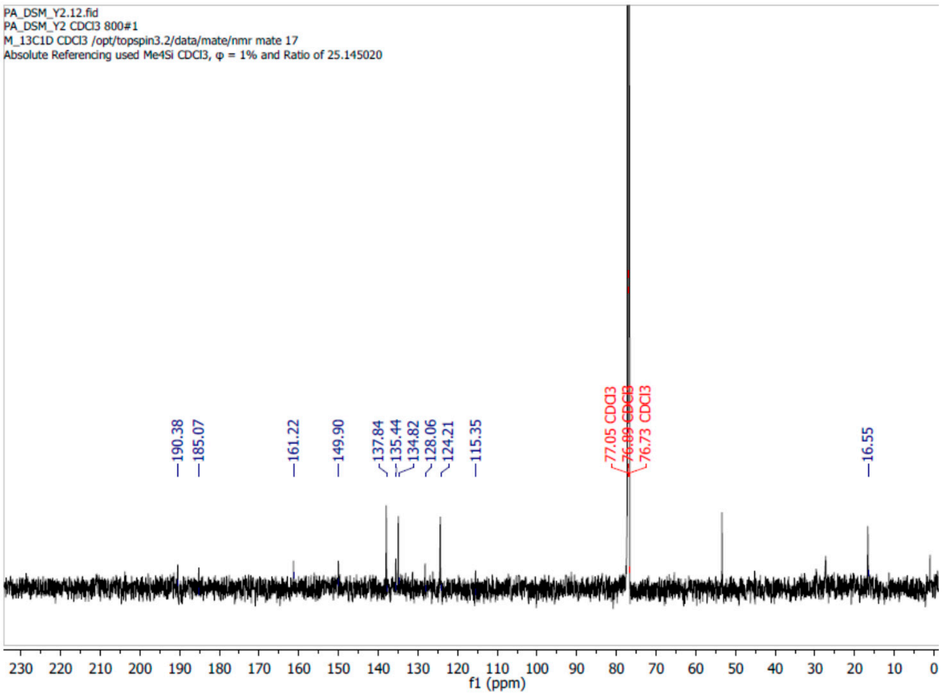

Figure S2. The ^{13}C-NMR spectrum of 8,8′-oxo-biplumbagin (**1**, $CDCl_3$, 25 °C, 201.20 MHz).

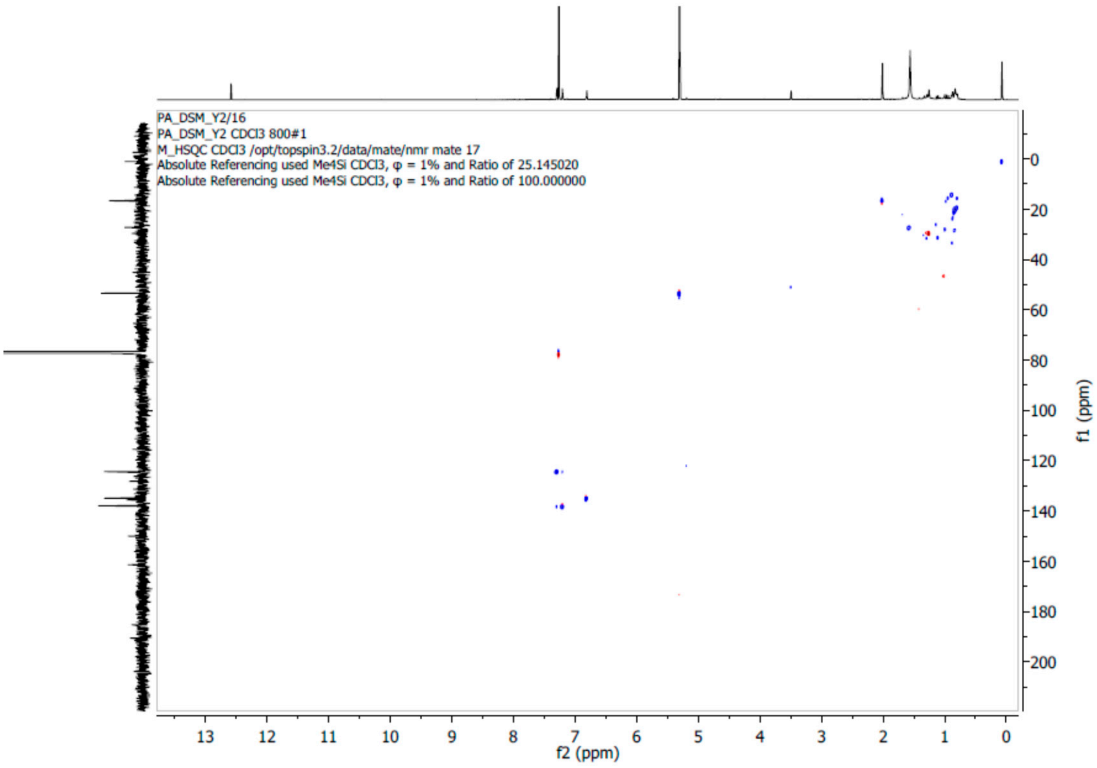

Figure S3. The ^{1}H-, ^{13}C-HSQC spectrum of 8,8′-oxo-biplumbagin (**1**, $CDCl_3$, 25 °C, 201.20 MHz).

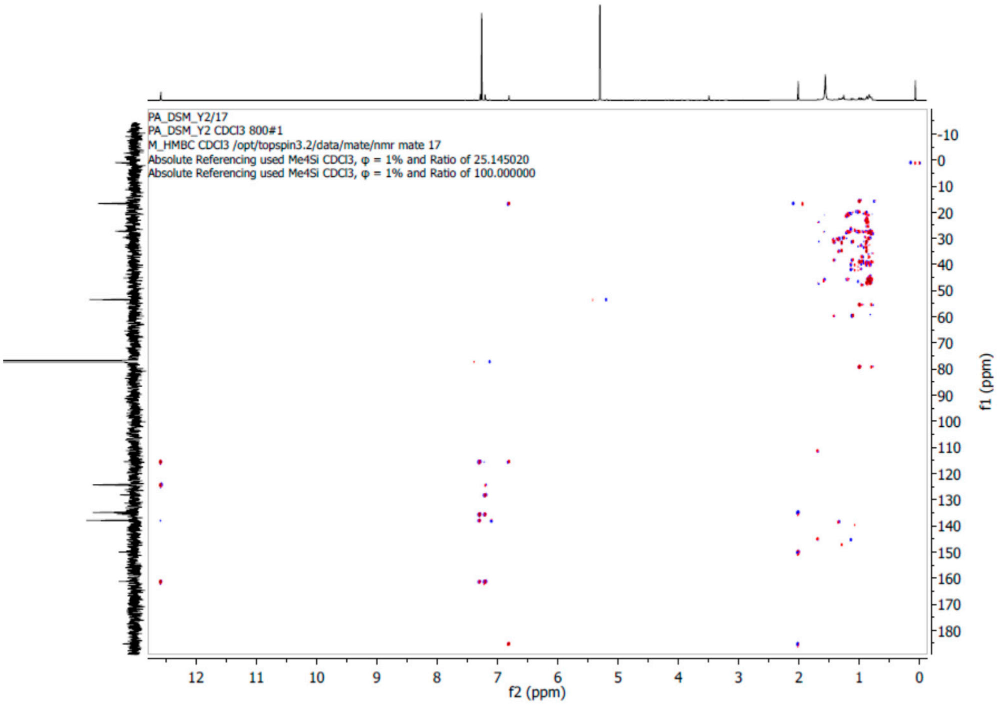

Figure S4. The ^{1}H-, ^{13}C-HMBC spectrum of 8,8′-Oxo-biplumbagin (**1**, $CDCl_3$, 25 °C, 201.20 MHz).

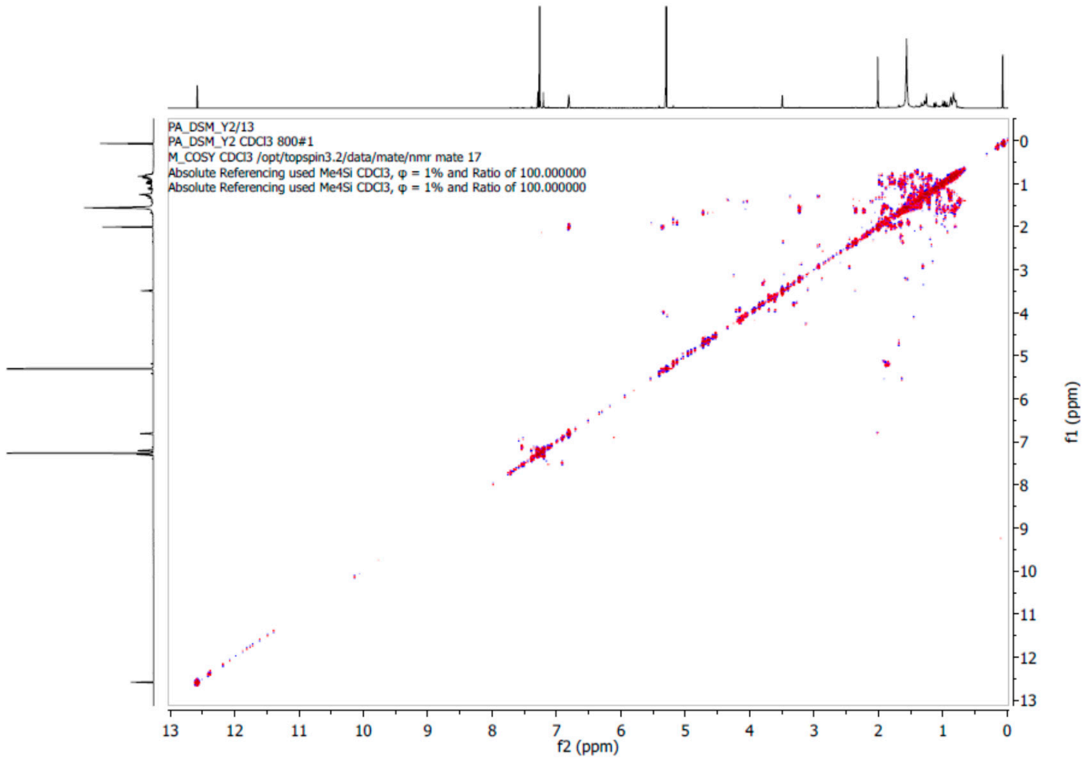

Figure S5. The ^{1}H-^{1}H COSY spectrum of 8,8′-oxo-biplumbagin (**1**, $CDCl_3$, 25 °C, 799.88 MHz).

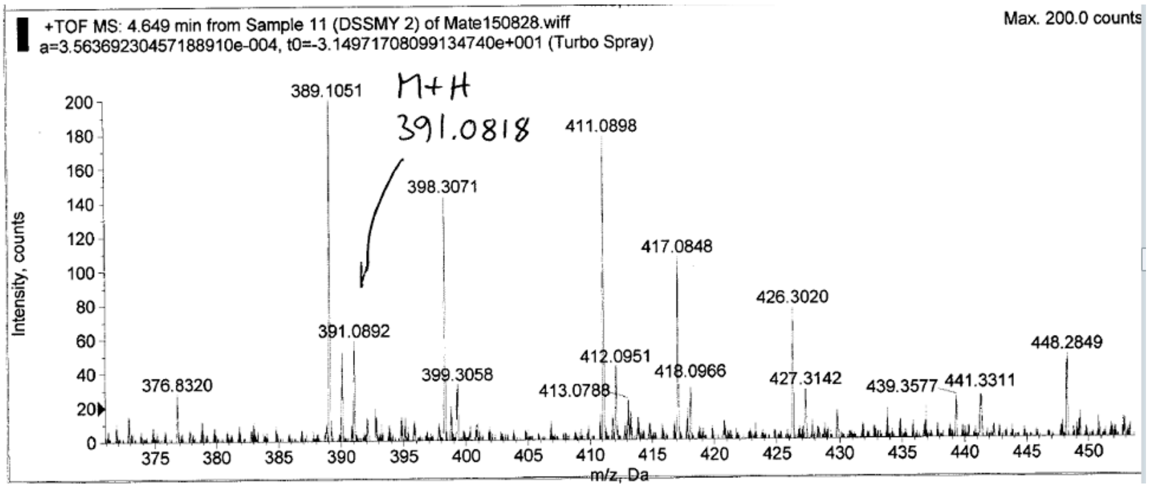

Figure S6. The HR (ESI) mass spectrum of 8,8′-oxobiplumbagin (**1**).

2. Spectroscopic Data of the Known Compounds 2–5

trans-Isoshinanolone (**2**) [1]. White amorphous solid. ^{1}H-NMR ($CDCl_3$, 25 °C) δ (799.88 MHz, ppm): 1.19 (3H, *d*, *J* = 6.6 Hz, C-11), 1.92 (1H, *d*, *J* = 6.6 Hz, 1-OH), 2.29 (1H, *dddq*, *J* = 10.1, 7.3, 6.6, 4.1 Hz, H-2), 2.44 (1H, *dd*, *J* = 17.4, 10.1 Hz, H-3a), 2.93 (1H, *dd*, *J* = 17.4, 4.2 Hz, H-3b), 4.51 (1H, *brt*, *J* = 6.9 Hz, H-1), 6.94 (1H, *dd*, *J* = 8.4, 0.9 Hz, H-6), 7.12 (1H, *t*, *J* = 8 Hz, H-8), 7.51 (1H, *dd*, *J* = 8.4, 7.6 Hz, H-7), 12.48 (1H, *s*, 5-OH); ^{13}C-NMR ($CDCl_3$, 25 °C) δ (201.20 MHz, ppm), 18.0 (C-11), 37.7 (C-2), 43.6 (C-3), 73.9 (C-1), 115.5 (C-10), 117.3 (C-8), 117.5 (C-6), 137.2 (C-7), 146.0 (C-9), 162.7 (C-5), 204.8 (C-4). IR ($CHCl_3$) cm^{-1}: 3620, 1617. GC-MS (70 eV): *m*/*z* 192 $[M]^+$ (100), 177 (25), 150 (45), 121 (90).

cis-Isoshinanolone (**3**) [28]. White amorphous solid. ^{1}H-NMR ($CDCl_3$, 25 °C) δ (799.88 MHz, ppm): 1.2 (3H, *d*, *J* = 6.9 Hz, 2-CH_3), 2,4 (1H, *dddq*, *J* = 11.0, 7.0, 4.2, 2.8 Hz, H-2), 2.9 (1H, *J* = 17.5 11.0·Hz, H-3a), 2.6 (1H, *dd*, *J* = 17.5, 4.3·Hz, H-3b), 4.8 (1H, *d*, *J* = 2.8 Hz, H-1), 6.9 (1H, *dd*, *J* = 8.5 1.1·Hz, H-6), 6.9 (1H, *d*, *J* = 7.4 Hz, H-8); 7.5 (1H, *dd*, *J* = 8.4, 7.4· Hz), 12.4 (1H, *s*, 5-OH). ^{13}C-NMR ($CDCl_3$, 25 °C) δ (201.20 MHz,

ppm): 16.3 (C-11), 34.6 (C-2), 40.9 (C-3), 71.3 (C-1), 115.1 (C-10), 118.4 (C-8), 118.8 (C-6), 137.1 (C-7), 145.1 (C-9), 162.9 (C-5), 204.9 (C-4). IR ($CHCl_3$) cm^{-1}: 3619, 1619. GC-MS (70 eV): *m/z* 192 $[M]^+$ (50), 177 (10), 150 (25), 121 (100).

Plumbagin (**4**) [21]. Yellow armophous solid. ^1H-NMR ($CDCl_3$, 25 °C) δ (799.88 MHz, ppm): 2.19 (3H, *d*, *J* = 1.6 Hz, 2-CH_3), 6.81 (1H, *q*, *J* = 1.6Hz, H-3), 7.25 (1H, *d*, *J* = 1.2 Hz, H-6), 7.60 (1H, *t*, *J* = 7.9 Hz, H-7), 7.64 (1H, *dd*, *J* = 7.5, ·1.2 Hz), 11.98 (1H, s, 5-OH). ^{13}C-NMR ($CDCl_3$, 25 °C) δ (201.20 MHz, ppm): 16.7 (C-11), 115.3 (C-10), 119.5 (C-8), 124.3 (C-6), 132.2 (C-9), 135.6 (C-3), 136.3 (C-7), 149.8 (C-2), 162.0 (C-5) 185.0 (C-1), 190.4 (C-4). IR ($CHCl_3$) cm^{-1}: 3611, 1584. GC-MS (70eV): *m/z* 188 $[M]^+$ (100), 173 (25), 131 (45), 92 (25).

3,3′-Biplumbagin (**5**) [21]. Yellow armophous solid. ^1H-NMR ($CDCl_3$, 25 °C) δ (799.88 MHz, ppm): 2.08 (6H, s, 2-CH_3), 7.30 (2H, *dd*, *J* = 1.3, 8.4 Hz, H-6 and H-6′), 7.67 (2H, *t*, *J* = 8.0 Hz, H-7 and H-7′), 7.77 (2H, *dd*, *J* = 1.3, 7.5 Hz, H-8 and H-8′), 11.81 (2H, *s*, 5-OH and 5′-OH). ^{13}C-NMR ($CDCl_3$, 25 °C) δ (201.20 MHz, ppm): 16.7 (C-11 and C-11′), 115.3 (C-10 and C-10′), 119.5 (C-8 and C-8′), 124.3 (C-6 and C-6′), 132.2 (C-9 and C-9′), 135.6 (C-3 and C-3′), 136.3(C-7 and C-7′), 149.8 (C-2 and C-2′), 161.3 (C-5 and C-5′), 184.9 (C-1 and C-1′), 190.4 (C-4 and C-4′). GC-MS (70eV): *m/z* 374 $[M]^+$ (96), 331 (47), 303 (75), 278 (47), 250 (47).

3. NAMFIS Analysis

Table S1. Monte Carlo conformational analysis for compounds **2** and **3**.

***trans*-Isoshinanolone (2)**	***cis*-Isoshinanolone (3)**
M σ% = 0.979D + 02	M σ% = 0.614D + 02
Conformation 1 = 10%	Conformation 1 = 88%
Conformation 2 = 81%	Conformation 2 = 12%
Conformation 3 = 1%	
Conformation 4 = 9%	

All other molar fractions are 0.00.

Tables S2. Best fit solution for compounds **2** and **3**.

***trans*-Isoshinanolone (2)**			***cis*-Isoshinanolone (3)**		
**Assign.**	**Exp.**	**Calc.**	**Assign.**	**Exp.**	**Calc.**
**Interproton distances (Å)**			**Interproton distances (Å)**		
H1-OH	3.40	3.23	H1-2	2.10	2.40
H1-H2	2.80	2.82	H1-Me	2.80	2.85
H3a—H1	2.80	2.79	H3a-H4	3.50	3.50
H3b-H2	2.30	2.47	H3a-Me	2.60	2.79
H3b-Me	3.00	2.86	H3b-Me	2.90	2.85
H3a-Me	2.80	2.75			
RMSD = 0.40			RMSD = 0.16		
**Coupling constants (Hz)**			**Coupling constants (Hz)**		
H1-H2	8.2	7.6	H1-H2	2.8	2.3
H2-H3a	4.6	4.1	H2-H3a	4.3	3.5
H2-H3b	10.1	10.4	H2-H3b	10.9	10.7
RMSD = 0.47			RMSD = 0.55		

4. Antioxidant Assay

Table S3. DPPH radical scavenging activity (RSA) of compounds isolated from *D. shimbaensis*.

% RSA	Concentration (μM)			
	**25.0**	**12.5**	**6.25**	**3.13**
Compound **1**	41.2	38.5	34.8	32.0
Compound **2**	43.9	42.8	32.4	31.7
Compound **3**	58.7	49.1	41.5	38.1
Compound **4**	40.5	36.8	36.4	32.9
Compound **5**	42.6	38.2	35.7	30.7

5. Cytotoxicity Assay

Table S4. Cytotoxicity data of the constituents of *D. shimbaensis*, and of its crude extracts suing various solvents. The data was obtained on a MDA-MB-231 breast cancer cell assay.

Compound	IC_{50} (μg/mL)	95% Confidence Interval
**2**	>100	-
**3**	50.11848	-
**4**	24.62663	1.897 to 258.9
**5**	30.68116	6.054 to 278.4
**DSLD** Crude	72.97427	-
**DSRM** Crude	>100	-
**DSLM** Crude	44.74559	24.03 to 697.8
**DSSM** Crude	59.56605	1.951 to 148502
**DSRH** Crude	16.1141	1.285 to 374.6
**DSRD** Crude	29.74992	8.371 to 133.2

DSSM (methanol crude extract from the stem barks); **DSRH** (isohexane crude extract from the root barks); **DSRD** (dichloromethane crude extract from the root barks); **DSRM** (methanol crude extract from the root barks); **DSLD** (dichloromethane crude extract from the leaves); **DSLM** (methanol crude extract from the leaves).

References

1. Bringmann, G.; Pfeifer, R.M.; Breuning, M.; Reichert, M.; Messer, K.; Schraut, M.; Tóth, G. Structure of *trans*-isoshinanolone in the crystal and in solution. *Z. Naturforsch*. **2004**, *59*, 100–105.
